# A case of traumatic uterine avulsion in pregnancy

**DOI:** 10.1016/j.tcr.2023.100920

**Published:** 2023-08-21

**Authors:** M.E. Chambers, A.B. Nguyen Pham, G.F. Milone, E. Licon, Y.A. Bakhta, K.K. Chan

**Affiliations:** University of California, Irvine, Department of Obstetrics & Gynecology, 3800 West Chapman Ave, Suite 3400, Orange, CA 92868, United States of America

**Keywords:** Uterine avulsion, Trauma in pregnancy, Uterine rupture

## Abstract

Complete uterine avulsion is an extremely rare complication of trauma sustained during pregnancy. We present the case of a 21-year-old nullipara at 16 weeks' gestation who was involved in a high-speed motor vehicle collision with subsequent fetal demise. Initially she was hemodynamically stable and demonstrated small amounts of intraabdominal free fluid, therefore multidisciplinary conservative measures were undertaken. However, as her condition worsened, she was taken for exploratory laparotomy, revealing complete gravid uterine avulsion at the level of the cervicoisthmic junction. Due to hemodynamic instability and concerns for retroperitoneal bleeding, a supracervical hysterectomy was performed. Although a rare occurrence, our case demonstrates the need for a high level of suspicion for uterine avulsion in certain cases of trauma in pregnancy. This highlights the false reassurance provided by stable vitals in a pregnant patient that may mask ongoing bleeding and development of hemorrhagic shock, the importance of interpreting different imaging modalities together when the cause of instability is unclear, and the utility of a multidisciplinary approach. While our patient underwent hysterectomy due to hemodynamic instability, it is unknown whether earlier investigation with laparoscopy to confirm uterine integrity may have circumvented this and allowed for fertility-sparing management. As such, our case encourages the utilization of early diagnostic laparoscopy if there is concern for uterine avulsion for the consideration of alternative surgical interventions for management.

## Introduction

There are cases reported in the literature regarding uterine injuries and lacerations sustained during trauma. Complete uterine avulsion is one of the rarest of these events, making its diagnosis and management challenging. We present a case of uterine avulsion following a motor vehicle accident involving a pregnant patient.

## Case

The patient is a 21-year-old nullipara at 16 weeks' gestation brought to the emergency department after a motor vehicle collision at 60–80 miles per hour. She was the restrained front-seat passenger and self-extricated following the collision. Initial vital signs were notable for a blood pressure of 92/39 mmHg and a maternal heart rate of 100 beats per minute. On physical exam, the abdomen was diffusely tender with positive seatbelt sign over the right anterior superior iliac spine without peritoneal signs. Labs demonstrated a hemoglobin of 9.9 g/dL. Focused Assessment with Sonography for Trauma (FAST) scan showed small volume intra-abdominal free fluid. Limited obstetric ultrasound demonstrated an intrauterine pregnancy at approximately 16 weeks' gestation without a fetal heart rate and near anhydramnios. The uterus appeared slightly dextro-rotated with clear boundaries discerned.

**Fig. 1:**
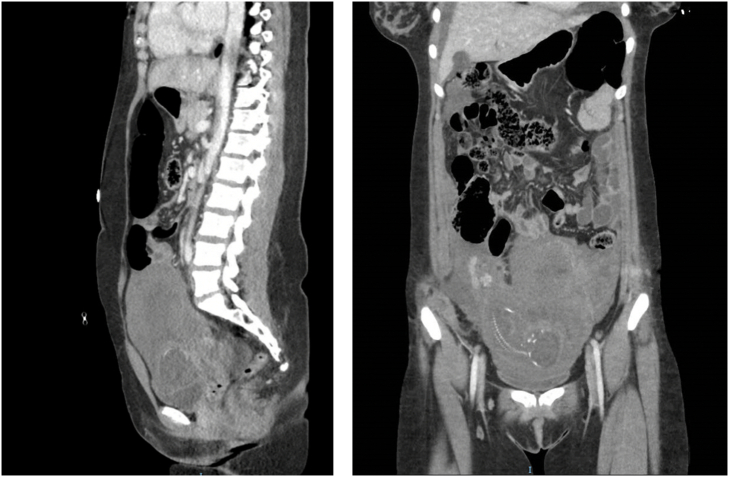
CT abdomen/pelvis in sagittal view demonstrating hemoperitoneum and unclear uterine boundaries in the sagittal (left) and coronal (right) views.

**Fig. 2:**
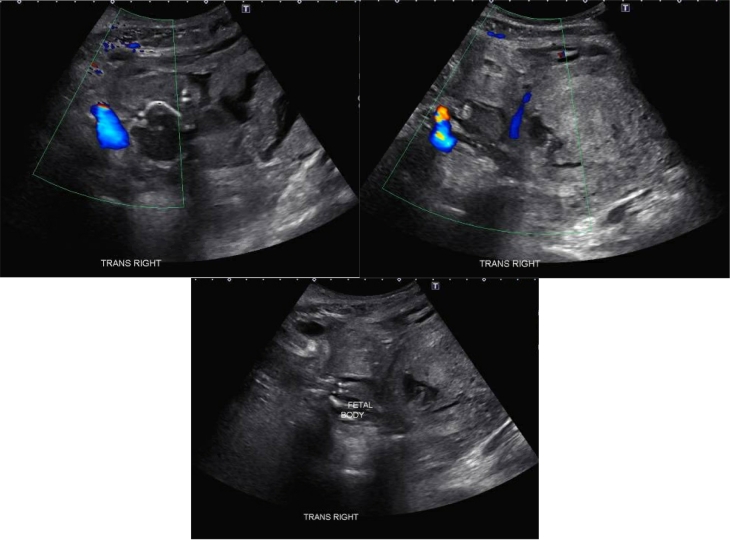
A. Transabdominal image of uterus adjacent to fetus with unclear identification of uterus wall. B. Transabdominal image of uterus without fetus inside uterus. C. Transabdominal image of fetus outside uterus.

A repeat FAST scan approximately 1 h later demonstrated increased pelvic free fluid. This was followed by a computerized tomography (CT) scan that showed large volume hemoperitoneum with inability to visualize the margins of the uterus, as seen in Fig. 1, Fig. 2. A focal laceration of the left common iliac artery was also found extending into the origin of the left external iliac artery.

Given the patient's continued hemodynamic stability despite these findings, a CT angiogram with embolization by Interventional Radiology was performed for management of the left common iliac artery laceration. A formal transvaginal ultrasound was then performed, and the right lateral wall of the uterus was unable to be visualized. When interpreting the CT and ultrasound images together, there was increased concern for traumatic uterine rupture. Additionally, a large clot adjacent to the bifurcation of the iliac arteries was increasing in size despite embolization. The obstetrics, trauma, and vascular surgical teams therefore jointly proceeded with exploratory laparotomy at this time.

**Fig. 3:**
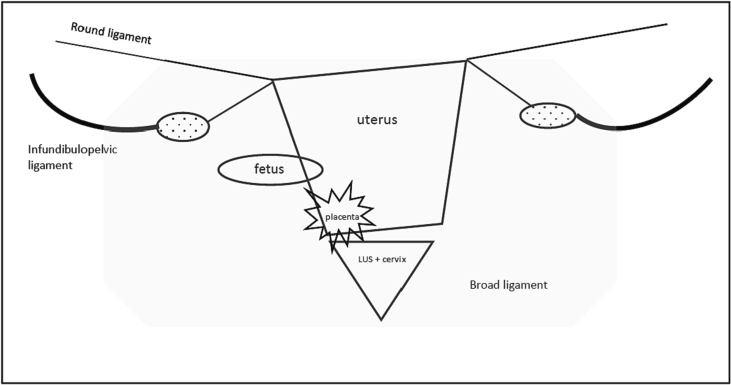
Diagram of intra-operative findings. Uterus avulsed from cervix and lower uterine segment (LUS), attached only by round ligament and infundipulopelvic ligaments. Fetus noted to be in the abdominal cavity with placenta partially detached.

Upon intraabdominal entry, three liters of hemoperitoneum were evacuated. The uterus was completely avulsed from the cervix at the level of the isthmus, tethered only by the broad, round, and infundibulopelvic ligaments bilaterally (Fig. 3). The demised fetus was found outside of the uterus. Due to complete cervico-isthmic disjunction and hemodynamic instability secondary to excessive intra-operative bleeding from major pelvic vessels, the decision was made to proceed with a supra-cervical hysterectomy. The bilateral ovaries were left in place. The vascular surgery team then performed a left iliac artery reconstruction and patch angioplasty with thromboendoarterectomy.

The patient was taken to the intensive care unit postoperatively. She was extubated on postoperative day two, transferred to the general medical floor, and discharged home in stable condition on postoperative day eight. A total of six units of packed red blood cells were transfused. The patient was advised that future fertility would require in vitro fertilization with a gestational carrier.

## Comment

Trauma occurs in approximately 8 % of pregnancies, with motor vehicle accidents as one of the most common causes, second only to domestic violence [[1], [2], [3]]. Injuries include obstetric and non-obstetric insults, with placental abruption being the most common obstetric complication, occurring in up to 40 % of major motor vehicle accidents [3]. Due to its unfortunate frequency, most obstetricians feel comfortable with the management of these cases.

Alternatively, a far less common complication of blunt abdominal trauma in pregnancy is uterine rupture, occurring in less than 1 % of cases, requiring providers to have a high index of suspicion [2,4]. Even more seldom encountered is the case we present of uterine avulsion with the complete separation of the uterine corpus from the cervix. As the isthmus is the weakest point of connection between the stronger uterine corpus and cervix, the point of disjunction is most seen at this region [5]. When the pelvis has been fractured, it is likely the crushing injury that impacts the uterine isthmus and amputates the corpus [6]. In cases without pelvic fracture, it is possible that trauma to the isthmus along the sacral promontory may lead to avulsion [6].

Regardless of mechanism of injury, the rarity of this event and the complexity of its presentation leaves providers with less guidance about management, highlighting the importance of providers sharing their experience in such cases.

### Diagnosis

The diagnosis of uterine rupture and complete avulsion is similar. However, the classic signs of uterine rupture in labor (abnormal fetal heart tracing pattern, abdominal pain, loss of fetal station, and maternal hemodynamic instability) are not always present following traumatic cases. Uterine rupture typically leads to intraabdominal hemorrhage. Other signs on abdominal imaging include unclear uterine borders and anhydramnios, suggesting uterine rupture and intraabdominal extravasation of amniotic fluid. Furthermore, uterine rupture not infrequently results in fetal demise, therefore loss of fetal heart tones should significantly raise suspicion.

In pregnancy, maternal blood volume increases by more than 50 %, allowing for hemodynamic protection against blood loss during delivery. Additionally, in the second trimester, heart rate increases and blood pressure decreases [2]. Collectively, these changes suggest that pregnant individuals may not manifest the typical signs of hypovolemic shock until 30–35 % of their circulating blood volume, or about two liters, has been lost [1].

Multidisciplinary care should be employed when treating a pregnant patient after a motor vehicle accident. As highlighted above, the pregnant patient differs dramatically from a physiologic standpoint compared to the non-gravid trauma patient. Identification of alternative warning signs is therefore prudent. While large volume free intra-abdominal fluid obviously suggests hemorrhage, this may take time to significantly manifest depending on the location of the injury.

### Operative management

As highlighted by our case and seen with others [4], patients with a traumatic uterine rupture may initially appear hemodynamically stable. Despite this, strong consideration should be made for early operative intervention if clinical suspicion is high. Regardless of fetal viability, early diagnostic laparoscopy should be considered to assess uterine integrity with conversion to laparotomy if needed. As maternal morbidity and fetal mortality rates are high with uterine rupture or avulsion [6,8], early detection may improve both outcomes. Further, even in the case of fetal demise, early intervention may allow for fertility sparing measures rather than necessitating hysterectomy. As reported by Suchecki et al., fetal survival is even possible depending on timing of intervention and location of uterine rupture [7].

While gravid uterine rupture often allows for uterine repair [4,7,8], to our knowledge, there are no cases in the literature of traumatic gravid uterine avulsion that have resulted in fertility sparing management [6,9]. In these cases, either the hemodynamic instability of the patient or the lack of integrity of vascular supply to the uterus has prompted the need for hysterectomy. Outside of pregnancy, however, there have been cases of traumatic uterine avulsion reported where hysterectomy was not necessary [5,[8], [9], [10]].

### Final comment

In our case, although the uterus likely retained vascular integrity, the patient was too hemodynamically unstable to proceed with such intervention. Not only was there continued active bleeding from her uterine injury, but beneath this she had an expanding hematoma from the left common iliac artery that necessitated urgent repair. Of note, this degree of pelvic vascular injury should always be considered in cases of severe trauma in pregnancy as significant pelvic venous congestion may increase the risk of retroperitoneal hemorrhage with pelvic injuries [1].

## Declaration of competing interest

The authors of this report have no conflicts of interest to be disclosed.

## References

[1] Sakamoto J., Michels C., Eisfelder B., Joshi N. (2019). Trauma in pregnancy. Emerg. Med. Clin. North Am..

[2] LA Rosa M., Loaiza S., Zambrano M.A., Escobar M.F. (2020). Trauma in pregnancy. Clin. Obstet. Gynecol..

[3] Mendez-Figueroa H., Dahlke J.D., Vrees R.A., Rouse D.J. (2013). Trauma in pregnancy: an updated systematic review. Am. J. Obstet. Gynecol..

[4] Dittrich K.C. (1996). Rupture of the gravid uterus secondary to motor vehicle trauma. J. Emerg. Med..

[5] Rashmi Suneja A., Yadav P., Sharma A., Vaid N.B. (2009). Uterine avulsion: a rare cause of cryptomenorrhea. J. Pediatr. Adolesc. Gynecol..

[6] Benijts G., Amy J.J., de Roose J. (1975). Traumatic avulsion of the pregnant uterus. Eur. J. Obstet. Gynecol. Reprod. Biol..

[7] Suchecki G., Tilden H., Roloff K., Chandwani D., Neeki M. (2020). Management of traumatic uterine rupture in blunt abdominal trauma: a case report and literature review. Cureus..

[8] Vignolle J., Lefebvre C., Lucot J.P., Rubod C. (2018). About a case of traumatic separation of the cervix from the uterine corpus, diagnosed in a context of infertility. J. Gynecol. Obstet. Hum. Reprod..

[9] Kesterson J., Dietrich J., Yussman M., Hertweck S.P. (2007). Secondary amenorrhea resulting from traumatic separation of the cervix from the uterine corpus. Obstet. Gynecol..

[10] Mankus E., Braun A., Knudtson J., Medrano-Valle G., McCann G. (2020). Immediate repair of a complete uterine transection after motor vehicle collision. Obstet. Gynecol..

